# Bone regeneration strategies based on organelle homeostasis of mesenchymal stem cells

**DOI:** 10.3389/fendo.2023.1151691

**Published:** 2023-03-17

**Authors:** Liangjing Xin, Yao Wen, Jinlin Song, Tao Chen, Qiming Zhai

**Affiliations:** ^1^ College of Stomatology, Chongqing Medical University, Chongqing, China; ^2^ Chongqing Key Laboratory for Oral Diseases and Biomedical Sciences, Chongqing Municipal Key Laboratory of Oral Biomedical Engineering of Higher Education, Chongqing, China

**Keywords:** mesenchymal stem cells, osteogenesis, subcellular homeostasis, target strategies, organelle regulation

## Abstract

The organelle modulation has emerged as a crucial contributor to the organismal homeostasis. The mesenchymal stem cells (MSCs), with their putative functions in maintaining the regeneration ability of adult tissues, have been identified as a major driver to underlie skeletal health. Bone is a structural and endocrine organ, in which the organelle regulation on mesenchymal stem cells (MSCs) function has most been discovered recently. Furthermore, potential treatments to control bone regeneration are developing using organelle-targeted techniques based on manipulating MSCs osteogenesis. In this review, we summarize the most current understanding of organelle regulation on MSCs in bone homeostasis, and to outline mechanistic insights as well as organelle-targeted approaches for accelerated bone regeneration.

## 1 Introduction

Developing from an intramembranous or endochondral ossification process, bone is an essential structural and endocrine organ, with highly metabolic process that undergoes continuous reconstruction from bone formation and resorption (1, 2). Bone associated mesenchymal stem cells (MSCs) have self-renewal and multi-directional differentiation potential and interact with the stem cell ecological niche extensively and dynamically. MSCs are considered to be core participants to tissue homeostasis and regeneration (3, 4). Notably, there are growing evidences that dysfunctions of MSCs, such as proliferation inhibition, osteogenic differentiation bias and disordered niche modulation, underlie the pathogenesis of inflammatory bone diseases, mainly including osteoporosis, osteoarthritis and rheumatic arthritis, and periodontitis, which have become the hotspots of bone research (5, 6). Currently, bone implant materials and nanomedicines are receiving significant attention in the treatment of inflammatory bone diseases due to their superior performance in controlling inflammation and guiding bone regeneration. However, due to the complex pathological microenvironment, implant materials and nanomedicines are difficult to reach subcellular targets and are prone to off-target effects, reducing bioavailability and efficacy severely (7, 8). Hence, the exploration of precision medicine for inflammatory bone diseases is of critical importance to public health and social welfare.

Building targeting strategies based on pathogenesis has become an established paradigm for the development of implants and delivery systems. The critical role of organelles in regulating the function of MSCs has been continuously revealed. Notably, organelles, such as mitochondria (9), lysosomes (10), endoplasmic reticulum (ER) (11), and the nucleus (12), have been implicated in the pathogenesis of stem cell impairment in bone homeostasis abnormalities (13). In the epoch of precision medicine, achieving precise organelle-targeting provides an ideal guiding paradigm for the restoration of homeostasis in MSCs and personalized treatment of inflammatory bone diseases. Organelle-targeted techniques can precisely govern therapeutic agents delivery from the plasma membrane to the site of action, improving drug efficiency while establishing the concentration required to evoke osteogenic differentiation of MSCs. Thus, organelle-targeted strategies have shown promising potential for overcoming physiological and pathological barriers, narrowing toxic side effects, and improving therapeutic efficacy to maximize the therapeutic effect. Here, we summarize the aspects of targeting structure, subcellular organelle function, organelle-mediated mechanisms of bone homeostasis, and design guidelines for advanced organelle targeting systems for MSCs.

## 2 Mitochondria: Biosynthetic and signaling transduction pumps

### 2.1 Structural and functional component of mitochondrion

With nearly 70 years of research progress, mitochondria have emerged as the most studied organelle in biomedical sciences (14). The understanding of mitochondria has evolved from the original cellular “energy factory” to a dynamic organelle for biosynthesis and signal transduction, enabling a shift from mitochondrial medicine to dynamic organelle-based precision medicine (15, 16). Mitochondria are associated with several complex cellular processes, from autophagy (17) to MSCs differentiation (18) and regulation of the immune response (19, 20). In addition to their intracellular role as organelles, mitochondria also perform physical transfers between cells and establish intercellular communication (21, 22). These findings weaken the cellular boundaries and instead emphasize the bidirectional transmission of biological information from organelle to organism.

The multifunctionality of mitochondria is parallel to the fact that they are enclosed by two membranes: the inner mitochondrial membrane (IMM) and the outer mitochondrial membrane (OMM), each of which has a different composition and function (20). The OMM serves as an interface separating the mitochondria from the cytoplasm, manages small molecule permeation processes, and mediates cellular signaling (23). Additionally, the OMM acts as a site of membrane contact for the exchange and reaction of components between mitochondria and other subcellular organelles (15), while the IMM presents as a highly folded structure that defines the mitochondrial matrix, providing an expansion of the surface area and containing a large number of shuttle ion channel transporter proteins and mitochondrial respiration complexes (24). The IMM is invaginated and shapes cristae, which is the key site of the oxidative phosphorylation pathway (OXPHOS) that control cellular respiration and energy conversion (25). The OXPHOS system consists of five enzyme complexes and two mobile electron carriers operating in the electron transport chain (ETC). The entire ETC synergistically drives the production of ATP, which is subsequently used as the primary energy carrier in almost all processes and is a central link in cellular metabolic processes (26). Studies have demonstrated that ETC supercomplexes (SCs) can assist in the assembly and stabilization of complex I, which together with complex III_2_ constitutes the main redox center of SCs and minimizes reactive oxygen species (ROS) production (27, 28). In addition, Kim HN et al. suggested that the pro-apoptotic and anti-osteoclastic effects of estrogen are induced by the inhibition of mitochondrial complex I activity in osteoclast progenitors (29). The intermembranous space (IMS) separates the OMM and IMM and serves as a pivotal buffer between the cytoplasm and the mitochondrial matrix (MM) (24), which is essential for metabolism and free radical scavenging (15, 30). MM participates in metabolic processes by mediating tricarboxylic acid (TCA) cycle, fatty acid oxidation and amino acid metabolism (31). In addition, MM is enriched with a large amount of mitochondrial DNA (mtDNA). Mitochondrial dysfunction is frequently caused by excessive mtDNA replications and mutations, which in turn set off various inflammatory cascades (32, 33). There is growing evidence that excessive mtDNA mutations appear as age-increasing characteristics and that they may be an integral part of the aging process, which in turn impairs osteogenic differentiation and leads to bone loss (34). Moreover, mitochondria play a significant role in regulating free calcium (Ca^2+^) and control a variety of Ca^2+^-dependent signaling networks (35). Going forward, linking bioenergetic processes of subcellular organelle to physiological, health-related biological phenotypes of mitochondria will greatly facilitate advances in the broader biomedical sciences.

### 2.2 Mitochondria: Housekeepers of controlling MSCs fate

**Figure 1:**
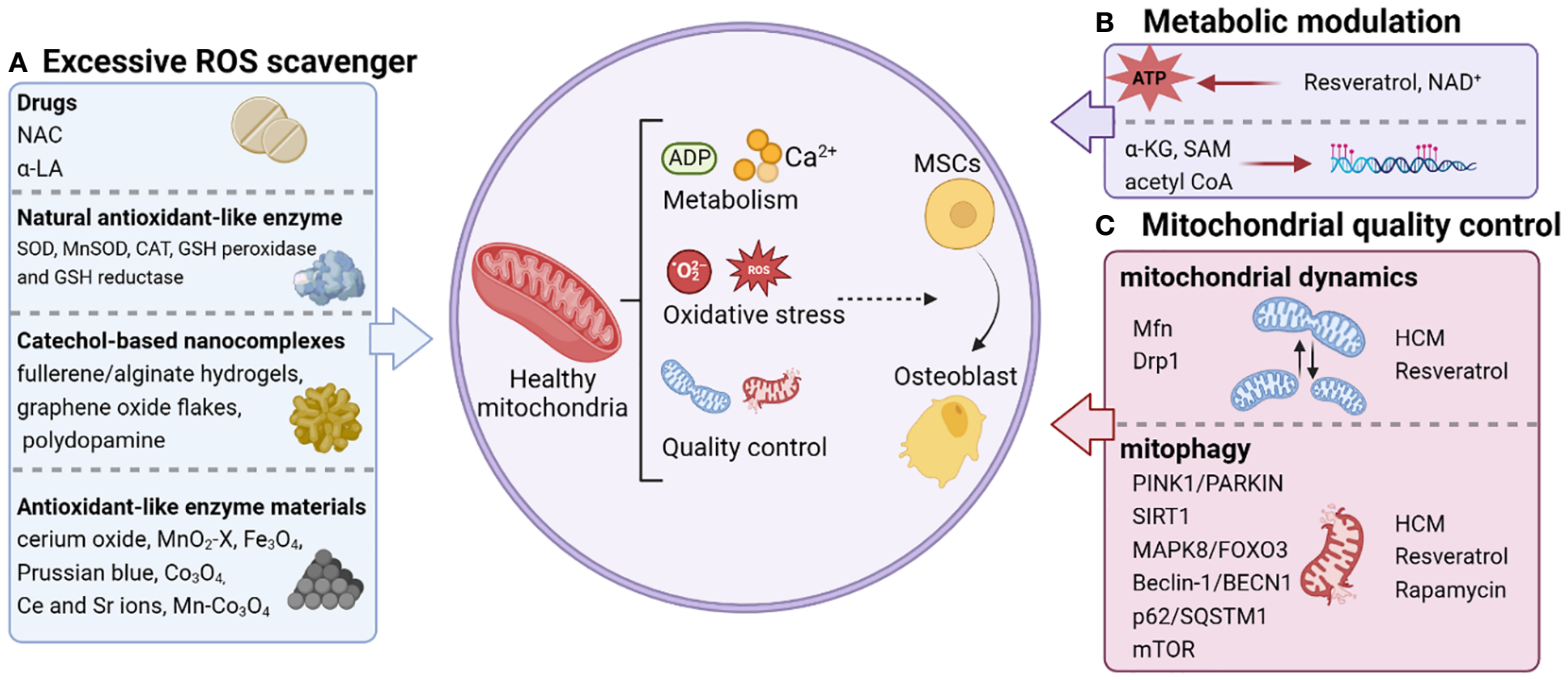
Personalized strategies for remodeling mitochondrial homeostasis. The regulation of mitochondrial function that triggers osteogenic differentiation of stem cells includes scavenging excessive ROS **(A)**, metabolic modulation **(B)**, and mitochondrial quality control **(C)**. NAC, N-acetylcysteine; α-LA, alpha-lipoic acid; SOD, superoxide dismutase; CAT, catalase; GSH, glutathione; MnSOD, manganese superoxide dismutase; Ce, cerium; Sr, strontium; NAD^+^, Nicotinamide adenine dinucleotide; α-KG, alpha-ketoglutarate; SAM, S-adenosylmethionine; acetyl CoA, acetyl coenzyme A; Mfn, Mitochondrial fusion protein; HCM, high molecular weight polyacrylic acid (HPAA)-crosslinked collagen membrane.

Mitochondria have emerged as key inputs for regulating essential cellular functions, such as metabolism, redox maintenance, Ca^2+^ homeostasis, and signal transduction (36). To maintain physiological functions, mitochondria actively reshape their morphology and function through redox regulation in conjunction with their metabolic and quality control mechanisms (9), which provides targets for precision medication (**Figure 1**).

The main pathway for producing ROS, which can invoke downstream phosphatases, kinases, and transcription factors, acting as crucial second messengers in MSCs self-renewal and differentiation, is mitochondrial respiration, which serves as the center of maintaining redox homeostasis (37–40). The maintenance of ROS at a stable level under physiological conditions plays an indispensable role in maintaining the proliferation and differentiation of different adult stem cells and MSCs (37). Excessive ROS accumulation, however, results in the loss of quiescence and apoptosis of stem cells (41, 42). The high levels of oxidative stress induced by excessive ROS make stem cells more susceptible to exogenous stimuli, leading to reduced stemness and suppressed osteogenic capacity (43, 44).

The mitochondrial metabolic profile, mainly referring to glycolysis and OXPHOS, not only meets the different metabolic demands of MSCs but also determines their fate and function. Through a variety of routes and enzymatic reprogramming, energy metabolism is switched from glycolysis in the cytosol to OXPHOS in the mitochondria during differentiating (45–48), which is especially crucial for the transformation of MSCs into osteoblasts. During osteoblast formation, oxygen consumption rates and intracellular ATP levels are remarkably increased, demonstrating the relevance of mitochondrial energy metabolism in the differentiation spectrum of MSCs (49–51). Inhibition of OXPHOS activity with respiratory chain complex inhibitors (e.g., antimycin A), uncoupling agents (e.g., FCCP) or ATP synthase inhibitors (e.g., oligomycin) increased mitochondrial membrane permeability and decreased membrane potential (MMP, Δψm) in MSCs, initiating stem cells senescence and apoptotic signaling (50, 52). Metabolites generated in mitochondrial OXPHOS have been confirmed to involved in the regulation of quiescence, self-renewal, and genealogical assignment of MSCs (53–55). Emerging evidence shed lights on that mitochondria depend on acetyl coenzyme A (acetyl CoA) provided by OXPHOS to activate the classical Wnt/β-catenin signaling pathway associated with bone formation, promote acetylation of β-catenin, stabilize and induce nuclear translocation, and increase the transcriptional activity of downstream master regulators (e.g., Runx2 and Osterix) of osteogenesis (56, 57). Accordingly, mitochondrial metabolites are substrates for a variety of chromatin-modifying enzymes that can regulate gene expression in MSCs by controlling chromatin modifications (methylation and demethylation of DNA and histones) (58, 59). It was demonstrated that alpha-ketoglutarate (α-KG)-dependent dioxygenases are regulated by demethylases (JHDMs) (45). In contrast, succinate, ferredoxin and 2-hydroxyglutarate, which are structurally similar to α-KG, competitively inhibit TETs and JHDMs, negatively regulating gene expression (60, 61). Furthermore, mitochondrial one-carbon metabolism (1CM) contributes to the maintenance of intracellular S-adenosylmethionine (SAM) pools generated in OXPHOS, is mediated by DNA methyltransferase 3a (Dnmt3a) to initiate histone and DNA methylation, and regulates RANKL-induced osteogenesis through epigenetic inhibition of the anti-osteoclast gene interferon regulatory factor 8 (IRF8) (62, 63). Recent studies have revealed that the N6-methyladenine (N6-mA) DNA modification can be regulated by SAM and α-KG and is essential for the maintenance of osteogenic differentiation in bone marrow-derived MSCs (BMSCs) (64, 65).

During the osteogenic differentiation of MSCs, morphological alterations of mitochondria are mainly manifested by enlargement and elongation, and increased volume (51). In contrast, the mitochondrial morphology in the inflammatory bone diseases is swollen and fragmented, requiring more energy through glycolysis compared to normal cells (66). This morphological alteration mediates mitochondrial regulatory mechanisms mainly dominated by quality control, including mitochondrial biogenesis, dynamism and mitophagy, which cross overlap and jointly determine mitochondrial function (67, 68). For example, mitochondrial transcription factor A (TFAM) has been identified to be closely associated with osteogenesis of MSCs. Overexpression of TFAM causes increased mitochondrial biogenesis, enhances the abundance of β-catenin and transcriptional activity, activating the Wnt signaling pathway-mediated osteogenic differentiation (69). Mitochondrial fusion protein 2 (Mfn2), a mitochondrial membrane protein that regulates mitochondrial fusion, is involved in mediating mitochondrial dynamics. It exhibits mitochondrial elongation during the early stages of osteogenesis and is accompanied by enhanced expression of Mfn2. Knockdown of Mfn2 leads to inhibition of mitochondrial fusion, alteration of the bioenergetic profile, and loss of osteogenic differentiation of MSCs (70). Moreover, evidence have shown that mitophagy plays an indispensable role in the maintenance of osteoblast and osteoclast homeostasis (71, 72). Mitophagy restores cellular homeostasis in the inflammatory microenvironment *via* reducing ROS production by damaged mitochondria, tightening additional energy supply, and producing ATP during degradation (73). Multiple mitophagy-related signaling pathways, including PINK1/PARKIN (74, 75), SIRT1 (76), MAPK8/FOXO3 (76, 77), Beclin-1/BECN1 (78), p62/SQSTM1 (79), and mammalian target of rapamycin (mTOR) (80), are involved in the regulation of osteoblastogenic-osteoclastogenic homeostasis. The evidence further revealed that mitophagy is inextricably linked to mitochondrial dynamics. Loss of Drp1 leads to functional mitochondrial dysfunction and the accumulation of damaged fragmented mitochondria, caused by a weakened mitophagy (81). Zhong et al. proposed that excessive mitochondrial division in pluripotent stem cells (PSCs) bridges driving mitochondrial dynamics and Ca^2+^ homeostasis by increasing cytoplasmic Ca^2+^ entry and CaMKII activity, leading to ubiquitin-mediated proteasomal degradation of β-Catenin, suggesting that the balance between mitochondrial fusion and division is critical for maintaining Ca^2+^ homeostasis (82). Hence, there is an urgent need to develop therapeutic strategies to realize mitochondrial quality control.

Going forward, considering the central role of redox balance, metabolic modulation and quality control in regulating bone homeostasis of MSCs, we focused on reviewing therapeutic strategies to promote bone formation based on mitochondrial homeostasis (**Figure 1**).

### 2.3 Strategies for restoring mitochondrial homeostasis

#### 2.3.1 Excessive ROS scavenger

Various antioxidants and natural enzymes can eliminate excessive ROS and involve in the regulation of oxidative signaling in the behavior of MSCs (**Figure 1A**). N-acetylcysteine (NAC) is currently recognized as the most commonly used agent to scavenge ROS, significantly alleviate multiple osteoporosis (83), and promote fracture healing in aged rats (84). When filling rat femur defects with collagen sponges containing autologous BMSCs, the NAC pre-treated group showed a considerable increase in new bone formation compared to the control group. Substantially, NAC pre-treated BMSCs promote bone regeneration by protecting the local implantation sites from oxidative damage (85). Alpha-lipoic acid (α-LA), another mitochondrial antioxidant, has been shown to promote osteogenic differentiation of MSCs and inhibit osteoclast formation in the treatment of osteoporosis (86, 87). The ROS detoxification system is composed of natural enzymes such as superoxide dismutase (SOD) (88), catalase (CAT) (89), glutathione (GSH) peroxidase and GSH reductase (90). Oxygen radicals are converted into hydrogen peroxide by SOD, and hydrogen peroxide is converted into water by CAT or GSH peroxidase for the purpose of detoxification. Knockdown of manganese superoxide dismutase (MnSOD) in MSCs leads to impaired osteogenic differentiation (49), whereas mice companied with deficiency of MnSOD suffer from oxidative stress and eventually develop osteoporosis (91). Currently, clinical trials in rheumatoid arthritis and osteoarthritis have shown that intra-articular injections of SOD are explored as a therapeutic treatment for inflammatory bone diseases (92, 93).

In recent years, graphene oxide flakes, fullerene/alginate hydrogels, polydopamine and other catechol-based nanocomplexes have been intensively investigated as antioxidant nanostructures for ROS scavenging to protect implanted stem cells (94–96). Meanwhile, materials with catalytic ROS scavenging activity have also been found to be alternative or even more effective strategies for regulating stem cell fate, attributed to their enzyme-like behavior (97, 98) (**Figure 1A**). A series of nanomaterials such as cerium oxide, MnO_2-x_, Fe_3_O_4_, Prussian blue, and their composites have good antioxidant-like enzyme activity with higher stability than natural enzymes (99). For the treatment of osteoporosis, Chen et al. constructed microenvironmentally responsive biofunctional metal-organic framework (bioMOF) coatings *in situ* by hydrothermal method on titanium surface through the coordination of p-hydroxydiphosphonate (PXBP) and cerium (Ce)/strontium (Sr) ions. Specifically, Ce ions can exhibit catalytic properties similar to CAT and SOD to break down ROS in MSCs and restore their mitochondrial function (100). In addition, several studies have demonstrated that Sr can promote CAT/SOD activity and regulate mitochondrial dynamics, thereby enhancing diabetic osseointegration (101). Thus, Ce and Sr ions could be used to synergistically mediate the recovery of mitochondrial dynamics in MSCs to restore function and enhance osteogenesis (102, 103). On this basis, a new strategy based on Mn atom substitution is reported in a recent study for the development of high-performance ROS scavengers with fast enzyme-like catalytic kinetics (104, 105). Among various metal oxides, Co_3_O_4_ is considered as one of the most promising candidates for catalytic ROS scavenging due to its high redox potential of Co^3+^/Co^2+^ (106, 107). Thanks to the redox-active nature of Co, two oxidation states (Co^2+^ and Co^3+^) can be easily manipulated by introducing charge transfer between the guest metal and the host metal (108). Modulating the Co^2+^/Co^3+^ ratio in Co_3_O_4_ to modulate the electronic structure of the catalytic center may provide new opportunities for the synthesis of multifaceted and efficient ROS scavenging metal oxides. Tian and his team (109) enhanced the intrinsic and broad-spectrum catalytic reactive oxygen scavenging activity of Co_3_O_4_ nanocrystals, named Mn-Co_3_O_4_, by modulating its electronic structure. Thus, Mn-Co_3_O_4_ could effectively protect MSCs from ROS attack and rescue their function of osteogenic differentiation. In conclusion, these findings reveal that ROS scavengers have promising prospects for improving the efficacy of stem cell therapy in inflammatory bone diseases.

#### 2.3.2 Metabolic modulation

Resveratrol is a natural polyphenolic compound that improves mitochondrial function and maintains metabolic homeostasis by increasing peroxisome proliferator-activated receptor γ coactivator-1 (PGC-1) activity (110, 111). It has been used to control, prevent, and reverse the devastating progression of inflammatory diseases, such as periodontal disease. Another study elucidated that resveratrol promotes bone differentiation in MSCs and resists age-related osteoporosis by initiating the Mitofilin or PGC-1α pathway to restore mitochondrial OXPHOS in MSCs (112).

In addition to the application of exogenous substances to produce a regulatory effect on mitochondrial metabolism, the direct repletion of metabolites opens a new therapeutic chapter. Nicotinamide adenine dinucleotide (NAD^+^), a crucial cofactor of OXPHOS, has been revealed to regulate the lineage commitment of BMSCs differentiation through OXPHOS (113). The results demonstrated that osteogenic committed BMSCs exhibited upregulated OXPHOS activity and diminished glycolysis accompanied by elevated intracellular NAD^+^ levels. On the contrary, an upregulated activity in glycolysis and resulted in a decline in NAD^+^ levels were observed in adipogenic committed BMSCs. The reduced NAD^+^ levels due to mitochondrial dysfunction and down-regulated OXPHOS activity significantly impaired the osteogenic differentiation of BMSCs. Administration of the NAD^+^ inhibitor FK866 delayed fracture healing *in vivo* (114). Collectively, NAD^+^-mediated mitochondrial OXPHOS is essential in osteogenic differentiation in BMSCs, and maintaining NAD^+^ levels become a novel therapeutic target for regenerative medicine. In this regard, Cho YS et al. demonstrated that supplementation with exogenous NAD^+^ delayed D-galactose (D-gal) induced BMSCs senescence and increased intracellular NAD^+^ levels (115). Silencing of Sirt1 exacerbated D-gal-induced senescence and attenuated the protective effect of exogenous NAD^+^ on senescent BMSCs. In the same way, the exogenous delivery of α-KG offers a novel option for the management of osteoporosis (116). As a key intermediate in the TCA cycle, α-KG regulates immune homeostasis and is an important source of synthetic amino acids and collagen (117). It has been demonstrated that administration of α-KG promoted skeletal development in growing rats (118). Consistently, α-KG is reported to ameliorate bone loss due to hormone deficiency (119, 120). α-KG was first applied directly to the treatment of osteoporosis by Yuan’s group in 2020 (116). In this study, supplementation of α-KG significantly reduced osteoporosis-induced bone loss and accelerated bone regeneration in aged mice. Administration of α-KG *in vitro* improved the proliferation, migration, and osteogenesis of MSCs by reducing the enrichment of H3K9me3 and H3K27me3 on BMP2, BMP4 and Nanog promoters. Taken together, supplementation of TCA cycle metabolites provides plausibility for potential therapies for the treatment of inflammatory bone diseases (**Figure 1B**).

#### 2.3.3 Mitochondrial quality control

Jiao and his team (121) found that upregulation of mitochondrial dynamics was closely associated with osteogenic differentiation of MSCs cultured on high molecular weight polyacrylic acid (HPAA)-crosslinked collagen membrane (HCM). When MSCs were cultured on HCM with an autophagy inhibitor, mitochondrial dynamics were significantly inhibited and osteogenic differentiation of MSCs was reduced. The results demonstrated that targeting of mitochondrial dynamics is a potential regulator of osteogenic differentiation in MSCs in response to ECM stiffness.

Resveratrol, a familiar antioxidant, rescues the decline in osteogenesis of BMSCs due to aging by upregulating the expression of inner membrane proteins of mitochondria (Mitofilin, a core component of the mitochondrial contact sites) that control morphological changes in mitochondria (112). In addition, resveratrol has been identified to activate the Sirt3-Foxo3a-PINK1-PARKIN-Mitochondrial fusion-fission-mitophagy signaling network, reducing ROS, and preserving GSH and reducing the aging phenotype (122).

Rapamycin acts as an mTOR inhibitor by activating mitophagy in order to participate in mitochondrial quality control (123, 124). Rapamycin has been reported to rescue osteoporosis caused by multiple pathologies in mice and restore osteogenesis in MSCs. Liu et al. proposed that blockade of the phosphatidylinositol 3-kinase (PI3K)/AKT/mTOR signaling cascade by rapamycin treatment could ameliorate alcohol-induced osteoporosis by rescuing impaired osteo/adipogenic lineage differentiation in BMSCs (125). Meanwhile, blockade of IL4/IL4Rα-mediated mTOR signaling pathway by rapamycin treatment improved osteogenic differentiation in BMSCs, thereby rescuing the osteopenia phenotype in Fibrillin-1 (FBN1)-deficient mice (126) (**Figure 1C**).

### 2.4 Smart nanosystem designed for mitochondria targeting

Since mitochondria are highly impermeable organelles in contrast to the nucleus, it is difficult to transport and permeate therapeutic substances *via* their double-membrane structure (127, 128). There is an urgent need to develop mitochondria-targeted nanoplatforms that can meet the therapeutic critical requirements. Only lipophilic cationic substances may pass through the mitochondrial membrane’s two layers, which have a negative IMM potential, and accumulate in the matrix of the mitochondria with opposing concentration gradients (129, 130). There are three classes of moieties including delocalized lipophilic cation, transition metal complexes and mitochondria-targeting peptides and sequences.

Although lipophilic cations have garnered a lot of interest and have been used in tumor therapy, stem cell research on them is still in its infancy. Triphenylphosphonium (TPP), gadolinium (DQA), berberine (BBR), rhodamine, and anthocyanin colors are examples of frequently used off-domain lipophilic cations (32, 131–133). Among these compounds, TPP is frequently employed in mitochondria-targeted nanosystem development. The Nernst equation states that TPP acts at MMP (at -180 mV) and hydrophobic sites on the mitochondrial membrane, and so that it can travel quickly through the membrane (133, 134). The engineered nanoparticles synthesized in our previous study conferred the ability to directly target mitochondria by grafting TPP on the surface of positively charged mesoporous silica nanoparticles (11). In the microenvironment of periodontitis and osteoarthritis, the grafted TPP nanoparticles have the ability to preferentially target the mitochondria of periodontal ligament stem cells (PDLSCs) and BMSCs, and precisely target the osteogenic differentiation of MSCs. We refer to the engineered nanoparticles as “mitochondrial repair agents”, whose specific responsiveness and selectivity allow all activities to occur only in diseased MSCs, thus achieving effective mitochondrial restoration without off-target effects, resulting in significant relief of periodontitis and osteoarthritis. Thus, off-domain lipophilic cations offer a promising strategy for the treatment of various chronic inflammation-associated bone diseases.

The utilization of peptide-based nanosystems, which enable the deliberate creation of peptide sequences or structural motifs as required, is an emerging method for attacking mitochondria. Horton et al. created the first mitochondrial penetrating peptide (MPP) after being inspired by cell-penetrating peptides (CPPs), and they showed how it facilitated cellular internalization and intra-mitochondrial localization (135). In addition to MPP, XJB peptides, Szeto-Schiller (SS) peptides, and ATAP peptides are also applied in mitochondria-targeted nanoplatforms (66). However, there are no studies related to stem cell therapy for inflammatory bone diseases.

## 3 Lysosomes: Initiating autophagy to regulate bone homeostasis

### 3.1 Structure and function

Since their discovery in the 1950s, lysosomes have been considered the “recycling stations” of cells, responsible for the degradation of a wide range of biological macromolecules (136). As the simplified understanding of the organelles has evolved, lysosomes are now defined as a dynamically regulated process that is a key determinant of cellular function (137). Each mammalian cell includes between 50 and 1000 lysosomes distributed in the cytoplasm, with a size of no more than 1 μm (138). The cell type and state have a significant impact on the form, size, and number of these characteristics. Lysosomes are single membrane-enclosed vesicles with a pH of 4.5-5.0 that are made up of 7–10 nm phospholipid bilayers, containing unique acidic lumens (139, 140). When biological macromolecules reach the lysosomes *via* various pathways, including endocytic, phagocytic and autophagic pathways, they are degraded in the lumen of the lysosomes by dozens of acidic hydrolases and subsequently by cellular metabolic processes reuse (137). Thus, lysosomes are a fundamental physiological link in cellular life activities and it is anticipated that they will become a new target for many disorders.

In a related lysosomal pathway, autophagy is a cellular response to stress. Chaperone-mediated autophagy, microautophagy, and macroautophagy are three kinds of autophagy with distinct regulation mechanisms (10). Macroautophagy is the most comprehensively researched of these because it is the most engaged in cell biology, physiology, and disease. Macroautophagy, also known as “autophagy”, is a process that maintains intracellular homeostasis by degrading and recycling intracellular metabolites, supplying energy and nutrients, and abolishing cytotoxic substances. In the classical autophagic pathway, cytoplasmic components are isolated in double-walled membrane vesicles that form autophagosomes. The whole process includes nucleation, extension and closure of vesicles or phagosomes (141). Following autophagosome formation, the lysosomal membrane and the autophagosome’s outer membrane fuse to produce an autophagic lysosome, which enables a variety of enzymes to break down the contents, including proteins, nucleic acids and lipids (142). ATG (autophagy-related) genes and ATG proteins are thought to be the central mechanisms of autophagosome biogenesis (143). During autophagosome nucleation, a macromolecular complex composed of class III phosphatidylinositol 3-kinase (PtdIns3K) and BECN1 (beclin 1) is recruited (144). Two ubiquitin-like systems, the ATG12-ATG5-ATG16L1 complex and MAP1LC3/LC3 (microtubule associated protein 1 light chain 3), are involved in the extension process. LC3 is cleaved by ATG4 to form cytoplasmic LC3- I. Subsequently, binding to phosphatidylethanolamine, ATG7-activated LC3-I is bound to the membrane to generate LC3-II (145). LC3-II binds tightly to phagosomes and autophagosomal membranes and is a typical marker of autophagosome completion, and therefore LC3-II protein is widely identified as an indicator of autophagy (146).

### 3.2 The effect of autophagy on bone regeneration

During bone remodeling, autophagy plays an important role in maintaining osteogenic-osteoblastic homeostasis by mediating immune regulation. It is indicated that autophagy plays a bidirectional regulatory role in promoting or inhibiting the osteogenesis process (71, 147). Yin et al. (148) reported that autophagy was suppressed in PDLSCs cultured under inflammatory conditions, and this inhibition of autophagic function was mediated by disorders of autophagosome-lysosome fusion and impaired activation of transcription factor EB (TFEB). Researchers have found that Lithium chloride inhibit apoptosis of MSCs in osteoporosis and reduces bone loss through upregulation of autophagic flux (149, 150). Conversely, in wear particle-induced osteolysis models *in vitro* and *in vivo*, implant wear particles (CoCrMo metal particles) over activate autophagy and inhibit bone formation, becoming the most common cause of implant aseptic loosening and total hip arthroplasty failure (71). Application of autophagy inhibitors significantly reduces wear particle-induced osteoblast apoptosis and ameliorates osteolysis *via* an anti-autophagic way. Proper activation of autophagy is a key pathway for cells to respond to the toxic response of biomaterials, and increased autophagic flux is a vital link for biomaterials to promote osteogenic differentiation of MSCs, providing a reasonable and feasible approach for designing therapeutic strategies to reshape bone homeostasis.

### 3.3 Lysosomes-targeted therapies for the recovery of MSC function

#### 3.3.1 Metals

Titanium, as the preferred choice for orthopedic and dental materials due to its superior biocompatibility and mechanical properties (151, 152). Researchers have utilized exosomes derived by macrophage stimulated with BMP2 to intrigue titanium oxide nanotubes to exert bone regeneration (153). The incorporation of BMP2/macrophage derived exosomes substantially upregulated the expression of osteoblastic differentiation markers in MSCs. Notably, the promotion of osteogenic differentiation by functionalized titanium oxide nanotubes was autophagy-mediated. For biomaterials, surface topography varies in order to interact better with the surrounding tissues. Titanium-based dental implants with rough surfaces induced osteoblast differentiation through autophagy-dependent PI3/Akt signaling pathway. The rough surfaces promoted the formation of cell clusters, which is important for the formation and mineralization of bone nodules. Once autophagy was inhibited, cell cluster formation was suppressed and osteogenic capacity is reduced (154). Similarly, titanium surfaces modified with nanotopographies had a higher osteogenic differentiation phenotype, initiating signaling between YAP and β-catenin that is autophagy-mediated (155). Collectively, the above data demonstrated that autophagy is indispensable for osteogenic differentiation of titanium-based rough surfaces. Silver, which has excellent antibacterial properties, is also widely used in medical treatment to delay and avoid bacterial infections (156). He et al. (157) investigated the effect of silver nanoparticles (AgNPs) on osteogenesis of MSCs and elucidated the potential mechanisms. The results illustrated that AgNPs upregulated the osteogenic protein expression and mineralization of MSCs. Meanwhile, the autophagic pathway was activated by AgNPs, whose induced osteogenesis was proportional to the autophagic flux, and the upregulated autophagic pathway was involved in the osteogenesis of MSCs induced by AgNPs. Numerous data suggested that the osteogenic potential of PDLSCs was impaired under inflammatory conditions, and gold nanoparticles (AuNPs) rescued the impaired osteogenic potential of PDLSCs by restoring the inflammation-impaired autophagosome-lysosome system. Knockdown of TFEB, a major regulator of the autophagy-lysosome system, prevented AuNPs from exerting its rescue effect on inflammatory PDLSCs, revealing a critical role of the autophagy-lysosome system in promoting osteogenesis of PDLSCs under inflammatory conditions (148) (**Table 1**).

**Table 1:** Targeting autophagy: A promising therapy for osteogenesis.

	Biomaterials	Autophagy Markers (up/down)	Autophagy Mechanism	Osteogenesis Marker (up/down)	Biological Effect	references
**Metals**	Titanium oxide nanotubes	LC3II/LC3I ↑ ATG5 ↑	PI3/Akt, YAP, β-catenin	ALP, BMP2, BMP7, Runx2, OCN, CoL1, OPN ↑	Activated autophagy during osteogenic differentiation	(146–148)
	Silver NPs	LC3-II ↑, p62 ↓		ALP, COL1, OPN, OCN↑	Stimulated collagen secretion, ECM mineralization; Autophagy and modulated osteoblast differentiation	(150)
	Gold NPs	LC3, Beclin-1 ↑ p62 ↓		ALP, RUNX2, COL1, OPN, OCN↑	Stimulated Mineralization, Autophagy and modulated osteoblast differentiation	(143)
**Ceramics**	Hydroxyapatite	LC3II/LC3I ↑	mTOR	ALP, BMP2, BSP, COL1, OSC, Runx2 ↑	Autophagy and modulated osteoblast differentiation	(152)
	Polydopaminetemplated Hydroxyapatite (tHA)	LC3B II, Beclin-1 ↑	AMPK/mTOR	OPN, Runx2, ALP activity, Alizarin red ↑	tHA combined with metformin regulated autophagy, improved the activity of hPDLSCs, and promoted osteogenic differentiation	(153)
	solid silica nanoparticles	LC3-II ↑, p-ERK/ERK ↑, p-AKT/mAKT, p-mTOR/mTOR ↓	ERK1/2, AKT/mTOR	ALP, mineralization level, COL1, OPG, OCN, OPN, and RUNX2 ↑	Enhanced the differentiation potential by enhancing autophagy	(154)
	Bioactive glass		AKT/mTOR	ALP, alizarin red S staining ↑	Improved autophagy, promoted osteogenic differentiation of OVX-BMSCs and bone regeneration in osteoporotic bone defects	(155)
	Strontium-doped BG	LC 3-II/LC 3-I, Beclin-1 ↑	AKT/mTOR	BMP-2, OPN, BSP, OCN ↑	Activated autophagy and osteogenic differentiation, bone mineralization and calcium deposition	(156)

(↑ stands for upregulated and ↓ stands for downregulated).

#### 3.3.2 Ceramics

Hydroxyapatite (HAP, Ca10(OH)2(PO4)6) has chemical properties similar to those of the inorganic components of the bone matrix and is a naturally occurring mineral found in the human skeleton. HAP offers significant advantages over other bone substitutes (e.g., allogeneic bone or metal implants) in clinical applications due to its enhanced binding to the host tissue (158). Previous studies have shown that different morphologies of HAP can activate autophagy in MSCs, thereby promoting vascular and bone regeneration (159). Scaffolds constructed with spherical nano-HAP can also promote osteogenic differentiation by modulating autophagy in a dose-dependent manner (159). Another study confirmed that polydopamine-templated hydroxyapatite (tHA), a nano-biomaterial that can replace conventional HAP, plays a crucial role in bone tissue engineering. High concentrations of tHA inhibited the expression of autophagy-related proteins beclin1 and LC3II in human PDLSCs (hPDLSCs), inducing excessive ROS production and leading to cell damage and apoptosis. Nevertheless, the combined application of tHA and metformin triggered the activation of autophagy by the AMPK/mTOR signaling pathway, thereby preventing the cytotoxicity of exposure to high concentrations of tHA and further enhancing the osteogenic effect of hPDLSCs (160). Chen and his group (161) investigated the biological effects of different silica nanomaterials (solid silica nanoparticles, mesoporous silica nanoparticles, and biodegradable mesoporous silica nanoparticles) on MSCs differentiation. Compared with the other two silica nanomaterials, solid silica nanoparticles upregulated the expression of LC3-II through ERK1/2 and AKT/mTOR signaling pathways, activated the autophagic flux of MSCs, and enhanced osteogenic differentiation potential. Essentially, it was found that solid silica nanoparticles had a low protein uptake capacity and could promote direct interaction of nucleolar nanoparticles. It will contribute to the future development of silica-based nanomaterials in translational medicine. Bioactive glass (BG), another porous silica-based nanomaterial, have also been widely applicated in bone regeneration. Patel et al. applied BG for surface modification of implants and demonstrated that BG can markedly enhance the osteogenic capacity of implant materials (162). Moreover, Sr-doped BG provided a promising strategy for promoting osteogenic differentiation and bone regeneration in osteoporotic bone defects through initiation of autophagy and activation of AKT/mTOR signaling pathway in BMSCs (163) (**Table 1**).

## 4 ER – targeted strategies: Remodeling protein homeostasis

### 4.1 Structure of the ER

Despite being one of the largest organelles in eukaryotic cells, the ER was among the last to be discovered (164). Since then, it has become clear that the ER is made up of a single continuous membrane that crosses the cytoplasm and connects to other organelles. The presence of ribosomes, cellular compartments responsible for protein translation and maturation, distinguishes the rough ER, which then fuse into transitional and smooth ER structural domains that perform a variety of essential cellular functions and play important roles, including protein folding, modification, export, regulation of cytosolic calcium homeostasis, lipid metabolism and cholesterol synthesis (165, 166). Only correctly folded peptides are transported to their destination after ER release because of the regulation of proper protein folding and complex formation by the protein mass monitoring mechanism found in the ER lumen (167, 168). Almost 30% of developing proteins are folded with the aid of a number of molecular chaperones in the ER lumen (167, 169). The occurrence of discrete domains between the ER and other organelles, known as membrane contact sites (MCSs), has received new attention in recent years. Organelle membranes are closely apposed and linked at these contact locations, but they do not fuse. Here, a range of protein complexes can collaborate to carry out specific tasks like binding, detecting, and transporting molecules, as well as participating in organelle formation and dynamics. More significantly, the ER controls the equilibrium of intracellular Ca^2+^ homeostasis as a Ca2^+^ storage compartment (170). It was first recognized that the prominent function of MCSs is to act as an important site for Ca^2+^ delivery and lipid exchange (171). Ca^2+^ is highly active in the cell and can act as a regulatory point for multiple signaling pathways. Ca^2+^ dysregulation or local abnormal accumulation can cause dysregulation of signal transduction and even cause cellular calcium toxicity (172). As the main site of Ca^2+^ and lipid storage, the ER is undoubtedly the biggest hotspot for research in this field.

Upon encountering an unfavorable stimulus, unfolded or incorrectly folded proteins have the ability to activate the unfolded protein response (UPR) signaling pathway, which then transports them out of the ER where they are degraded by the proteasome (173, 174). Disturbances in ER homeostasis may result in severe ER stress (ERS) if unfolded or misfolded proteins are not promptly cleared (175). Recently, there has been growing proof suggesting the UPR is essential for the survival and preservation of stem cells (11, 173). As the largest intracellular “protein workshop”, the ER links the dynamic balance of the entire intracellular subcellular organelles, and there is a tremendous need to develop ER targeting strategies to enhance the significance of inflammatory bone disease therapy.

### 4.2 ERS: Enemies or friends? It depends.

**Figure 2:**
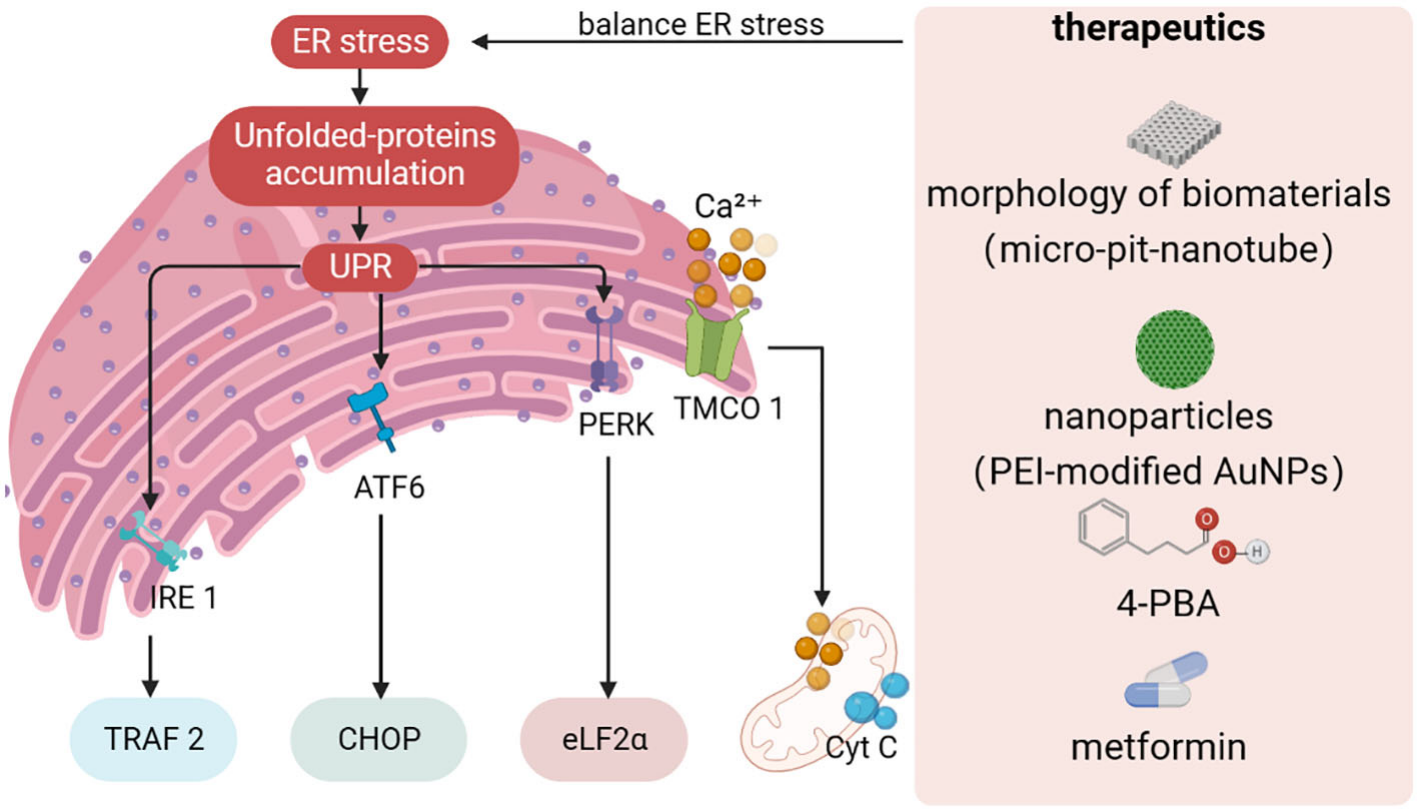
Design strategies towards to ER stress. Protein misfolding or unfolding can cause disorders in ER homeostasis, leading to ER stress. If ER stress is not resolved in time, unfolded or misfolded proteins accumulate in the ER, and the UPR triggers a cascade through IRE1α, ATF6, and PERK signaling pathways. At the same time, fluctuations in ER and mitochondrial Ca^2+^ homeostasis can trigger interactions between organelles. The establishment of therapeutic strategies for ER stress has become an indispensable part of regulating ER homeostasis to promote osteogenic differentiation. UPR, unfolded protein response; TMCO1, transmembrane and helix-coil structural domain 1, 4-PBA: 4-phenylbutiric acid.

The imbalance between the unfolded proteins load entering the ER and the cellular mechanisms that deal with the ER load leads to three major motor responses to the UPR effect. First, a transient adaptation achieved by reducing protein synthesis and translocation capacity into the ER mediates a reduction in protein load into the ER. Second, a long-term adaptation mechanism activated by transcription of UPR target genes increases the ability of the ER to process unfolded proteins. If endostasis cannot be re-established, a third mechanism, cell death, is triggered (176). Three different types of ERS sensors have been identified. Three transmembrane proteins-mediated signaling (PERK, IRE1α and ATF6) have been identified as ERS and contribute to the pro-survival pathway (177, 178) (**Figure 2**). To date, there is growing evidence that the UPR pathway may be a central regulatory system of signal transduction during osteogenic differentiation (179–181). Specifically, PERK plays an important role in neonatal skeletal development by regulating osteoblast proliferation, differentiation and type I collagen secretion (182). In contrast, in the absence of ATF4, bone formation is delayed and abnormal, and osteogenic protein expression is significantly reduced (183). A recent study showed that nanoparticles can induce ERS, which leads to apoptosis (184). Several studies have confirmed that the fate of MSCs or osteoblasts is closely related to the intensity of ERS, which is similar to mechanical stimulation. Researchers have found that long-term proinflammatory cytokines in periodontitis induce persistent ERS and reduce the osteogenic differentiation capacity of PDLSCs. Interestingly, chronic inflammation leads to the upregulation of the key UPR sensor PERK by downregulating the expression of histone acetyltransferase lysine acetyltransferase 6B (known as MORF), which leads to the sustained activation of UPR in PDLSCs and aggravates the inhibition of osteogenic differentiation *in vivo* and *in vitro* (185). Excessive ERS may lead to apoptosis, whereas compatibility forces can accelerate bone formation *in vitro* and *in vivo*.

Likewise, Ca^2+^ in the ER plays an integral role in ERS-mediated dysfunction (**Figure 2**). Intracellular Ca^2+^ signaling has been shown to play a critical role in maintaining various cellular functions such as proliferation and osteogenic differentiation (186, 187). Disruption of ER Ca^2+^homeostasis may cause abnormal fluctuations in cytoplasmic Ca^2+^ concentration, which in turn leads to stem cell dysfunction. When ER Ca^2+^ flux and leakage occur, large amounts of Ca^2+^ can enter and accumulate along mitochondria-associated membranes (MAMs), disrupting mitochondrial function (188). Thus, how to narrow down the excessive ERS effect and keep it within the adjustable range to regulate the potential of stem cells for osteogenic differentiation has become an important issue to be addressed.

### 4.3 Targeted therapeutic strategies in response to ERS

It is well known that both the morphology of biomaterials and the UPR pathway can significantly affect the osteogenic differentiation of stem cells and controlling UPR-mediated osteogenic effects by modulating the surface morphology of biomaterials is expected to be an emerging therapeutic strategy (189, 190) (**Figure 2**). Shi et al. prepared micro-pit-nanotube morphology on pure titanium foil by etching and anodic oxidation in hydrofluoric acid and systematically investigated the relationship between ERS and the UPR pathway (191). It was found that ERS and PERK-eIF2α-ATF4 pathway were activated in a time- and morphology-dependent manner on the micro-pit-nanotube structures. The activation of UPR by various morphologies was consistent with their osteogenic induction ability. Furthermore, mild ERS facilitated osteogenesis, whereas fierce ERS impaired osteogenic differentiation and led to apoptosis. Taken together, the ERS state depends on the morphology of the cell attachment surface, which may indicate new insights into topographic signaling.

Several studies have confirmed that drugs and nanoparticles can accelerate the osteogenic capacity of MSCs (**Figure 2**). 4-phenylbutiric acid (4-PBA), proposed by Deborah Krakow, improves the phenotype including osteogenesis and calcium mineralization by attenuating the expression of UPR markers (including HSPA5, XBP1, ATF4, DDIT3 and ATF6) in stem cells. Application of 4-PBA is expected to be an effective strategy to suppress excessive ERS and promote osteogenic differentiation (192). Recently, Zhong et al. (193) found that metformin can rescue the function of impaired PDLSCs in the diabetic periodontitis setting by activating the transmembrane and helix-coil structural domain 1 (TMCO1) signaling axis, which has been shown to be associated with osteoblast differentiation and function by preventing ER Ca^2+^ overload. Studies have shown that hyperglycemia may lead to disruption of ER homeostasis through ER Ca^2+^ overload, and metformin may alleviate this disruption. Therefore, metformin is expected to be a first-line therapeutic agent for ERS overload caused by diabetic -mediated Ca^2+^ overload. Pan and co-workers (194) synthesized PEI-modified AuNPs that can effectively transduce miR-29b directly into MSCs, thereby inducing osteogenic differentiation. Interestingly, AuNPs enter the cytoplasm and are mainly dispersed in the lumen of the ER. AuNPs loaded into the ER exert stress on the organelle, causing moderate ERS and thus affecting the synthesis of bone-associated proteins.

### 4.4 Targeting strategies of ER

Targeted delivery of therapeutic drugs to the ER is vital for the treatment of inflammatory bone disorders because the ER governs stem cell osteogenic differentiation. Unfortunately, due to ER’s intricate structure, which includes a vast 3D interconnected network of various thicknesses, targeted navigation to the ER for therapeutic drugs is a tricky task (195). Currently, ER targeting strategies such as small molecules and metal complexes and ER-targeted peptides have provided key targets for cancer treatment progression (196). However, there has not yet been an application in MSCs.

Sulfonamide ligands have been thoroughly investigated for small molecule drug delivery vehicle modifications because of their low toxicity, outstanding effectiveness, and high selectivity (197). They detect and attach to high-affinity sulfonylurea receptors, which are potassium-selective ion channel proteins abundantly expressed on ER membranes. Similarly, peptides with the C-terminal sequence Lys-Asp-Glu-Leu motifs of KDEL (binding to KDEL receptors on ER membranes) or AAKKKA (peptide interactions with specific ER membrane proteins) are also capable of ER targeting (197–199). We believe that ER targeting of stem cells to enable osteogenesis will also be realized in the near future.

## 5 The nucleus: A genome-associated dynamic network for precision targeted strategies

### 5.1 Structure of nucleus

The nucleus, the living hallmark of eukaryotic cells, is the housekeeper of the majority genomes, playing a crucial role in maintaining the stability of genetic materials and regulating osteogenesis and metabolism (200). The latest findings proposed that the nucleus is no longer a simple rigid framework, but a dynamic organelle with unique substructure (200–203).

The nucleus is structurally and functionally composed of two parts: the nuclear envelope and the nuclear interior. The nuclear envelope is a dense network of proteins consisting of two phospholipid bilayers, divided into the nuclear membranes and the nuclear lamina, which dominates the physicochemical properties of the nuclear envelope (204, 205). Acts as a boundary, the nuclear envelope separates the nucleus from the cytoplasm, ensuring the exchange of macromolecules and the stability of genetic materials (206). The double nuclear membrane is divided into the inner and the outer nuclear membrane, which is distributed with numerous nuclear pore complexes and is an indispensable site for mediating bi-directional nucleoplasmic transport of biological signals (207, 208). The nuclear pore complex is a highly symmetric scaffold, whose center crosses the nuclear envelope as a bridging channel. Moreover, dozens of different nuclear pore proteins with biochemical stability are arranged within the building blocks of the nuclear pore complex (209). Nevertheless, the efficiency of nucleoplasmic transport is influenced by the physical properties of biomolecules. Relatively small molecules less than 40 kDa can passively diffuse through the nuclear pore complex, while macromolecules of more than ∼40 to 60 kDa are impeded. They achieve rapid transport must rely on energy- and signal-dependent transport processes mediated by nuclear import and export proteins. The malfunction of nucleocytoplasmic transport can lead to mislocalization of proteins, which affect gene expression and signal transduction (210–212).

With the rise of emerging detection technologies, significant advances in understanding the dynamic nucleoskeleton have been achieved in recent years. It is now obvious that the nucleoskeleton assigns the nucleus and the genome certain forms, mechanical properties, and functionality. The nuclear skeleton is the dominant element in the skeletal networks and its measured stiffness is about ten times more than the cytoskeleton. The nuclear laminas, which make up the majority of the nucleoskeleton and are necessary for the mammalian cytoskeleton (213, 214). The lamins are arranged in the inner nuclear membrane, conferring mechanical stability to protein and chromatin binding and providing a platform for entanglement, which act as mediators for a variety of nuclear processes, such as DNA replication and repair, chromatin control, transcription, and genome organization (215, 216). Lamins A and C, produced by alternative splicing of the LMNA gene, and lamins B1 and B2/B3, encoded by LMNB1 and LMNB2, respectively, are expressed by mammalian somatic cells. The nuclear interior houses chromatin and other subnuclear structure that plays an equally essential role in controlling cellular biological processes and determining mechanical properties (217). Therefore, changes in chromatin structure and organization have a direct impact on the mechanical properties the nucleus and nucleoskeletal structures can further affect the their effective stiffness (218). Taken it together, modulation of nucleoskeleton of MSCs for remodeling bone morphology and ameliorating bone structural defect caused by inflammatory bone diseases is the key to achieve nuclear-targeted therapies.

### 5.2 Nucleus and osteogenesis: From sensing microenvironmental signals to gene modulation

The nucleus is a fundamental link in guiding the osteogenic differentiation of MSCs by regulating the dynamics of the nucleoskeleton from macroscopic perception of the cell microenvironment to microscopic mediation of the modification and expression of genetic materials (219).

On the one hand, as a dynamic mechanosensor, the deformation of the nucleus due to the topography or mechanical stress of its culture environment can regulate gene expression, which is particularly essential for the functional reprogramming of MSCs (220, 221). Controlling the reprogramming of MSCs through morphology has promising applications in the field of tissue engineering and regenerative medicine. The topography of the environment is an element parameter controlling the biological behavior of MSCs (222). Topographical patterns change the shape of cells and force their cytoskeletons to rearrange, which has an impact on cellular and nuclear mechanics (223). MSCs are physically stimulated by biomaterials in a way that mimics key elements of the physiological environment, which in turn regulates their biological behavior (224). Meanwhile, biomaterials such as micropores, scaffolds or hydrogels can form cellular-scale environments that influence MSCs osteogenic differentiation by modulating the nucleoskeleton through altering matrix stiffness, topology and pore size (225–227).

On the other hand, as the largest cellular gene pool, the nucleus coordinates gene regulation by initiating a series of genetic mechanisms to maintain the homeostasis of MSCs. However, in pathological settings, large amounts of DNA are under attack (228). In order to maintain the osteogenic differentiation ability of MSCs, RNA modification, long non-coding RNA regulation, and DNA damage repair are particularly important.

### 5.3 Biomaterial topography regulates osteogenic differentiation strategies

#### 5.3.1 Matrix stiffness

**Figure 3:**
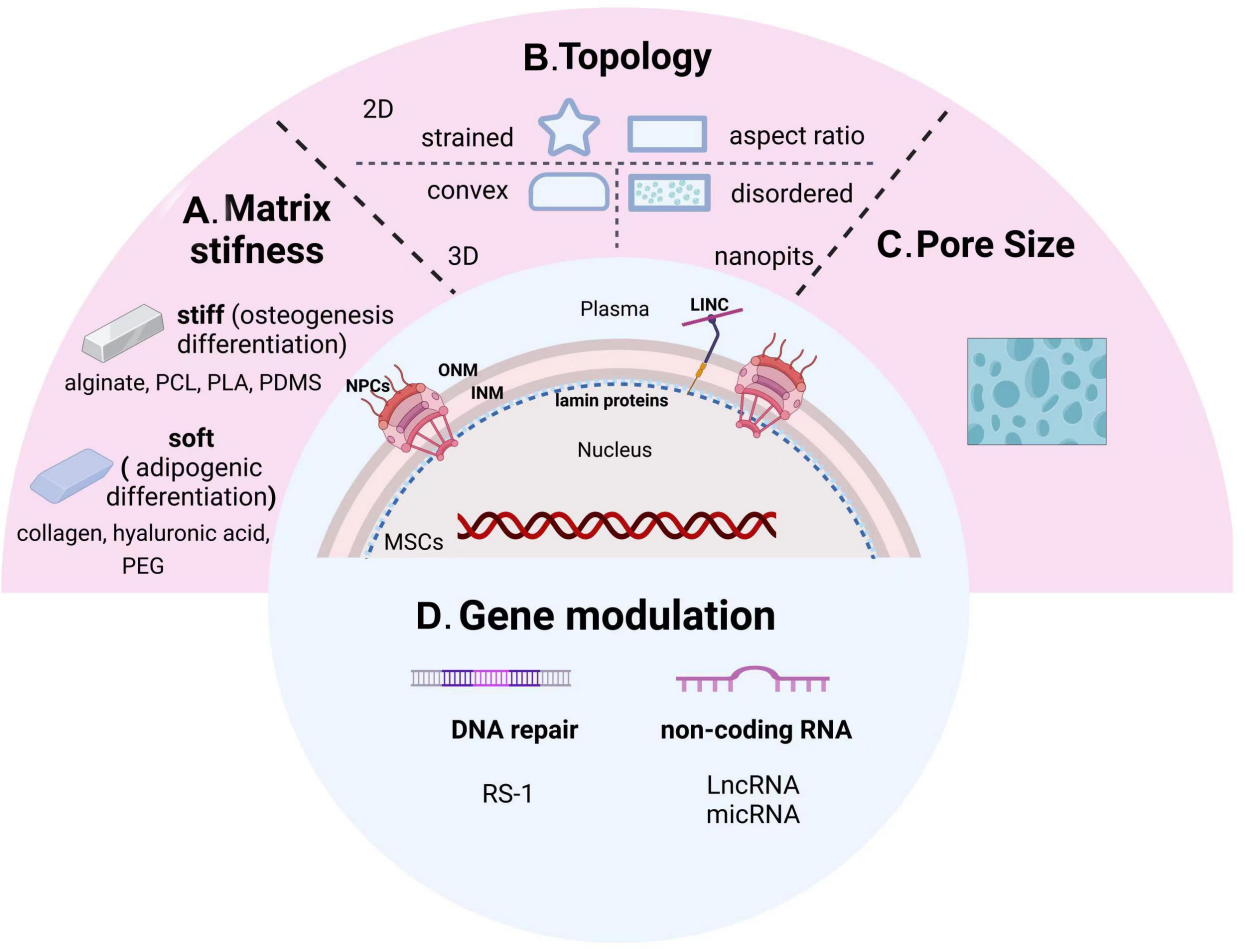
From sensing microenvironmental signals to gene modulation of nuclear-targeted guidelines. Physicochemical cues including matrix stiffness **(A)**, topology **(B)**, pore size **(C)**, and nuclear gene modifications **(D)** manipulate the osteogenic differentiation tendency of MSCs. PCL, polycaprolactone; PLA, polylactic acid; PDMS, polydimethylsiloxane; PEG, poly (ethylene glycol); INM, inner nuclear membrane; ONM, outer nuclear membrane; NPCs, nuclear pore complexes.

In order to better create a microenvironment suitable for cell growth, natural proteins or synthetic polymers meet the demand through changes in composition, concentration, and synthesis steps. Among them, the stiffness change of the scaffolds has a significant difference in guiding the directional differentiation of stem cells (**Figure 3A**). Hydrogels such as collagen, hyaluronic acid, and poly (ethylene glycol) (PEG) are generally soft materials with a stiffness below 800 Pa, which can be rapidly increased by adjusting their cross-linking density. These soft scaffolds are commonly used for adipogenic differentiation of stem cells. On the contrary, hard scaffolds with stiffness over 10kPa, such as alginate, polycaprolactone (PCL), polylactic acid (PLA), and polydimethylsiloxane (PDMS), are more suitable for bone remodeling (229).

Other studies showed that YAP/TAZ was predominantly cytoplasmic expressed on soft substrates, while nuclear translocation was promoted on hard substrates by modifying the stiffness of acrylamide hydrogels *via* addition of fibronectin. In addition, knockdown of YAP/TAZ enabled MSCs to differentiate into adipocytes on a hard substrate (230). However, overexpression of intranuclear TAZ could bind to Runx2 and perform robust osteogenesis in adipose-derived MSCs (231).

#### 5.3.2 Topology

*2D pattern:* Previously, 2D adhesive patterns of different sizes have been demonstrated to have the ability to control MSCs stem cell lineage (**Figure 3B**). It was found that the potential for osteogenic differentiation of human MSCs (hMSCs) was positively correlated with the state of adhesion, flattening, and spreading, while unspread round cells turned into adipocytes (232). Next, Kilian et al. demonstrated that the balance between osteogenesis and adipogenesis changed when MSCs were cultured on a rectangular surface with adjustable aspect ratio or on a shape with pentagonal symmetry but with different subcellular curvature. Further experiments showed that strained 2D patterns promoted osteogenic differentiation of MSCs by upregulating actomyosin contractility, initiating the activation of c-Jun N-terminal kinase (JNK) and extracellular related kinase (ERK1/2), and Wnt signaling pathway (233). Meanwhile, due to the development of micro- and nano-patterning technology, Peng et al. fabricated arginine-glycine-aspartic acid (RGD) micropatterns on PEG hydrogel to observe the effects of different micropatterned surfaces on MSCs differentiation. The aspect ratios of the micropattern plays a key role in MSCs differentiation, and its osteogenic and adipogenic differentiation exhibit different trends. The optimal aspect ratio (AR) for adipogenic differentiation is 1, while the ideal AR for osteogenic differentiation is roughly 2, which is based on the comparison of square and rectangular cells. Compared to the square and triangular star cells, the optimal adipogenic and osteogenic differentiations took place in circular and star cells, respectively (234). According to the aforementioned findings, adipogenesis and osteogenic differentiation levels were significantly correlated to cell perimeter.

*Nanopits:* By using different nanopits as models to alter cell morphology, Dalby et al. proposed that the ability to alter morphology and interphase nuclear organization in response to mechanical stress is the key scientific question in cell biology (235). MCSs cultured on surfaces with slightly disordered nanopits exhibited an increase in focal adhesion size and upregulation of osteopontin (224). MSCs accomplished self-renewal on the surface of ordered nanopits, demonstrating the possibility of controlling the osteogenic differentiation of MSCs by managing the disorder of nanopits (236) (**Figure 3B**).

*3D patterns:* Substrate curvature is involved in regulating fundamental cellular biomechanical processes, including adhesion, membrane protrusion and tension, cytoskeletal polymerization, and contraction, to control cell fate (**Figure 3B**). The researchers developed 3D mechanical models of single cell migration with different curvatures, and the mechanics of cells on the curved surface were evaluated by modeling. It was shown that hMSCs migrate more consistently on concave than on convex surfaces (237), and the nuclear structure is more stable and round (238). Further analysis of the shape and protrusion force of the cells on the substrate different curvatures suggested an altered migration pattern of hMSCs. Despite the fact that the cells spread out less on concave surfaces, the protrusion force magnitude in the direction of migration was larger than on convex ones. Hence, the substrate topography determines the direction of the protrusion force and promotes the continuous migration of the concave surfaces. Again, osteocalcin was found to be more expressed on convex surfaces compared to concave ones, suggesting the potential of convex features to promote osteogenic differentiation of MSCs (239).

#### 5.3.3 Pore size

The choice of pore size for the corresponding culture scaffold is different for stem cells from different sources (**Figure 3C**). The researchers applied melt electro writing to create scaffolds with varying pore sizes and different cells to examine the impact of pore size on cell fate. The evidence suggested that different cells adhered and proliferated at different rates on the scaffolds. Furthermore, the pore size of the scaffold also influenced cell differentiation and gene expression patterns. Among them, BMSCs showed the greatest viability on 200-μm pore size scaffolds, chondrocytes on 200-and 100-μm scaffolds, and tendon stem cells on 300-μm scaffolds (240). Dense and hard tissues such as bone and skin need to grow attached to smaller pore sizes, while cartilage and fat differentiations prefer scaffolds with larger pore sizes (241). This is due to the general belief that bulk porosity is negatively correlated with stiffness. The larger pore size sacrifices part of the solid material and produces a larger porosity, leading to weaker scaffold stiffness.

### 5.4 Gene modulation therapy

DNA damage induced by excessive ROS induces inappropriate transcriptional activation, resulting in the inability of stem cells to perform a range of essential physiological activities and greatly reducing the efficiency of osteogenic differentiation. Herein, maintaining genomic stability of the nucleus is crucial for the treatment of inflammatory bone diseases. Recently, the inhibitory effect of RS-1 (3-(benzylaminosulfonyl)-4-bromo-N-(4-bromophenyl) benzamide) on DNA damage during PEI-mediated gene therapy was explored (242). RS-1 has been implicated as a stimulator of RAD51 recombinase (RAD51), a key activator of proteins necessary for homologous recombination (HR) in the DNA repair process. Delivery of pRunx2 into hMSCs using RS-1 significantly reduced DNA damage and upregulated osteogenic differentiation potential (**Figure 3D**).

It was demonstrated that a long non-coding RNA (LncRNA) regulates the contact between the promoter and enhancer of the ECM protein fibromodulin (FMOD), which controls the fate of BMSCs during aging through local chromatin remodeling. For instance, lncRNA-Bmncr acts as a scaffold to promote the interaction between TAZ and ABL, thereby accelerating TAZ and assembly of the RUNX2/PPARG transcriptional complex. Knockout Bmncr mice showed reduced bone mass and increased bone marrow fat accumulation, while overexpression of Bmncr rescued the bone mass loss (243). Moreover, miRNA also participates the process of MSCs osteogenesis. It has been reported that the treatment of miR-26a effectively enhanced osteogenesis of MSCs isolated from osteoporotic mice, making it a promising therapeutic candidate for osteoporosis (244). Osteogenic differentiation is negatively controlled in mouse BMSCs by miR-338-3p, which directly affects Runx2, and may potentially be a factor in osteoporosis, which was demonstrated to be more prevalent in ovariectomized (OVX) mice compared to control animals (245) (**Figure 3D**).

## 6 Conclusion and outlook

With the rapid development of biomedicine, precise organelles-targeted therapies have become a hot topic in tissue regeneration. We discuss four primary organelles in our review-the nucleus, mitochondria, lysosomes, and ER- and briefly describe the distinctive characteristics and roles of each organelle in order to highlight their historic significance as therapeutic targets to provide guiding principles for the construction of delivery systems. Although regulated distribution at the organelle level has been accomplished, it is still rough to realize clinical translation. For inflammatory bone diseases, exploiting the underlying pathological mechanisms and developing new molecular targets are still tricky. Additionally, the efficacy and safety of organelle-targeted therapies need to be further investigated. In recent years, osteoimmunomodulation is an important concept targeting the interaction between immune cells and osteogenesis-related cells, emphasizing the intrinsic link between the immune system and the bone regeneration sequence. Strategies to modulate the osteoimmunomodulation will also be a key component of bone regeneration therapy in the future. As a result, in order to validate their translation into clinical applications, long-term monitoring of safety and efficacy as well as the development of experimental primate models are necessary. In summary, we emphasize the significance of organelle-targeted management strategies for MSC-based bone regeneration, which is crucial for the establishment of organelle-targeted materials and their medical translation.

## Author contributions

LX wrote the manuscript. YW participated in the literature search and related data sorting. QZ, TC and JS conceived and revised the manuscript. All authors read and approved the submitted version. All authors contributed to the article and approved the submitted version.
